# A Novel Theory in Regard to an Additional Function of the Liver

**Published:** 1888-02

**Authors:** F. A. Schmitt

**Affiliations:** Schulenburg, Texas


### A NOVEL THEORY IN REGARD TO AN ADDITIONAL FUNCTION OF THE LIVER.

*By F. A. Schmitt, M. D., Schulenburg, Texas.*

For Daniel's Texas Medical Journal.

IN a late German Journal, I find an article peculiarly novel, which it will perhaps interest your readers to become acquainted with; ideas some of which strike me as being exceptionally sound and worthy of consideration.

Allow me, therefore, the privilege of giving an exact translation, and space in your Journal for same.

The view advanced of late, that all infections and many, if not all diseases are atributable to bactaria or otherwise termed microorganisms—having found access into the system from without and which induce morbidity by multiplying with rapidity and preying on the system for their support—will to some extent justify the prevalent idea among the laiety, and among a goodly number of M. D's also, of the curative powers of drugs, and which is given expression in the German proverb :

Gegen den Tod ist kein Kraut gewachsen—or put into English—there is no plant extant with which death can be cured ; giving expression to the idea, that death is a disease in itself, differing in so far only from other ailments—as it is incurable.

All other disease, according to this simple reasoning, yield to remedies if properly selected, compounded and administered, and he among a simple minded class is the physician, par excellance, who has the most drugs at his command wherewith to wage war and conquor disease.

For this reason the patent medicine vendors of our day—who loudly proclaim to have discovered specifics for certain or all ailments, can rely on a profitable business as long as they are backed by conspicuously placed and properly distributed advertisements; if besides they have given their nostrums big, unpronounceable and incomprehensible names and can produce documents—mainly of the clergy—to testify as to the intrinsic value and curative properties of their respective hodgepodges.

Undoubtedly are we in possession of medicaments endowed with curative powers; I need but mention quinine in malarial fevers, mercury in syphilis and iron in chlorosis.

With "trivial" propriety some of the profession argue that, as we have found remedies with which we can successfully combat these affections, we may in the future discover other drugs for other diseases endowed with the same specific powers. If they, however, will closely investigate, if they will minutely look into the modus opperandi of the action of these drugs—they will find that with these so-called specifics even are we but assisting nature in curing; in the first two mentioned ailments, by aiding the natural powers in the destruction and elimination of morbid material from the system by artificially introducing elements antagonistic to this morbid matter, and in the other—chlorosis—by imparting to the blood—either in bulk or in nutriments—one of its essential but failing constituents and necessary stimulus—iron.

Only a few of the laiety, and not many of the profession are sufficiently enlightened to see in the physician but an aid, and much less in the surgeon, but a helping hand of the great power of nature—*vis medicatrix naturæ—;* for the surgeon who will attentively listen to the dictates of this great natural power, will use his appliances and the knife, only as "adjuvants" to natures own resources in the alleviation and cure of abnormalities.

In coaptating a fractured bone for instance or in amputating a diseased limb, removing a morbid growth, or in ligating a bleeding vessel, he is only assisting nature in her endeavor to mend these abnormalities; for nature will weld the fractured ends of a broken bone, will (eventually) shed the diseased limb or morbid growth, or close a severed artery; but natures efforts as far as regards "juxtaposition" of fractured (or dislocated) bones may be and generally are futile, and its process of ridding itself of morbid excrescences, diseased limbs, or in arresting hemorrhage often too slow, to admit of the benefits to be derived therefrom.

It is then that our devine gift of reasoning comes into play in executing natures designs properly and effectually. The same is true in internal medication: Nature, constantly striving to overcome abnormal conditions, needs but to be rightly understood and her endeavors skillfully aided. The physician will then accomplish as much in curing diseases as the surgeon who (abruptly) with artful appliances or the knife, cuts short natures endeavors ends.

But to elucidate still further, and for the sake of argument he continues, "we will admit that we in the distant future may discover the drug (Germicide) for all diseases producing germs," and having learned to use these agents without materially injuring an infested system, still he claims, that we are but assisting nature in her endeavor to rid itself of its numerous microscopical intruders, for unassisted nature has ways and means and avenues for their shedding.

I conceive, he says, two organs in particular, well adapted for the performance of this task.

He hypothetically assumes that the liver, while receiving through the portal system the blood in rapid succession, may, in conjunction with its other functions, rid the blood of its pathological impurities and is provided by nature with an avenue for their escape; (the gall ducts leading into the bowels) and on the other hand he says, may the skin perform its additional office as an eliminator by taking up (absorbing) morbid particles at the periphery and exuding them in diaphoresis, as indeed is claimed by Sir James Grant in diptheria and which (diaphoresis), is often the closing scene of febrile conditions.

The critical sweat of old authors, thus finds its physiological explanation, i. e. by reducing temperature and by eliminating from the system the inciting cause (morbid matter) wholly or in part.

So he concludes, until the time has arrived when we have discovered all the instigators of disease and their respective antidotes, it will be proper to assist nature as best we can by relieving the system of pathological impureties with the present means at our command by stimulating a healthy action of liver* and skin, and to bring about a general condition best suited to withstand the ravages of minute, but numerous enemies, all ot which may be accomplished if properly attended to.

It is not the remedy or remedies with which we cure, he again asserts, but it is as it ever was, the exact interpretation of the language of nature which should determine our action in regard to treatment, and he is the ideal physician (or surgeon), who fully understands, comprehends and skillfully interprets the idiom of nature.

* This is verified in malarial toxæmia certanly, as often a malarial attack may be verted by the timely administration of a dose of calomal or blue mass.

